# The Malignant Transformation Rate of Oral Carcinoma In Situ

**DOI:** 10.1002/lary.70025

**Published:** 2025-08-09

**Authors:** Andrew Meci, Neerav Goyal, David Goldenberg

**Affiliations:** ^1^ The Pennsylvania State University College of Medicine Hershey Pennsylvania USA; ^2^ Penn State Milton S. Hershey Medical Center Hershey Pennsylvania USA

**Keywords:** carcinoma in situ, cohort study, head and neck cancer, malignant transformation, oral cavity cancer

## Abstract

**Objectives:**

Oral carcinoma in situ (OCIS) is a Stage 0 malignant condition known to progress to invasive oral squamous cell carcinoma (OSCC). The rate and timeframe of this transformation are not well delineated. We present the largest study to date investigating the malignant transformation rate of OCIS to invasive OSCC.

**Methods:**

We performed a retrospective cohort analysis using the TriNetX Research database. Patients ≥ 18 years of age with a diagnosis of OCIS from 2008 to 2018 were included, allowing for up to 5 years of follow‐up. The primary outcome was the 5‐year rate of OSCC transformation compared to an age‐matched control cohort. Secondary objectives included a description of treatment pathways and 5‐year survival. Descriptive and comparative statistics were conducted using TriNetX software.

**Results:**

About 4130 patients diagnosed with OCIS were included. Mean age at diagnosis was 62.5 ± 13.1 with patients being 61.0% male (*n* = 2521), 74.0% white (*n* = 3058), and 73.3% not Hispanic/Latino (*n* = 175). Patients with OCIS had 15.3% nicotine dependence (*n* = 15.3) and 7.5% alcohol dependence (*n* = 308). The 5‐year malignant transformation rate for OCIS to OSCC was 56.8% (*n* = 2349), with the mean time to transformation being 15.0 months. Patients had a 234.9 times greater risk of developing OSCC compared to the control (95% CI 126.4–436.5, *p* < 0.01). Five‐year Kaplan–Meier survival probability for OCIS was 74.7%.

**Conclusions:**

Our results indicate a high rate of malignant transformation from OCIS to OSCC. A mean transformation time of 15 months underscores the need for prompt work‐up and treatment.

**Level of Evidence:**

Retrospective cohort study—Level 3.

## Graphical abstract

Oral carcinoma in situ (OCIS), where cancer cells are confined to the epithelium, is a Stage 0 malignant condition known to progress to invasive oral squamous cell carcinoma (OSCC). The rate and timeframe of this transformation are not well delineated. We present the largest study to date investigating the malignant transformation rate of OCIS to invasive OSCC.

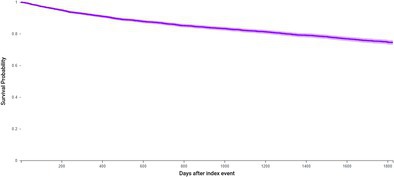

## 1 Introduction

Oral cavity squamous cell carcinoma (OSCC) is a group of malignancies involving the lip, buccal mucosa, gingiva, alveolar ridge, floor of the mouth, anterior tongue, retromolar trigone, and hard palate [1]. In the United States, the prevalence of OSCC has risen steadily while the age‐adjusted incidence rate of OSCC has decreased slightly over time; however, this finding is variable according to sex and other demographic characteristics [2]. OSCC may arise de novo but is often preceded by pre‐cancerous and pre‐invasive conditions, which, if treated promptly or intensively monitored, may improve OSCC outcomes or even prevent OSCC from arising [3, 4].

Oral carcinoma in situ (OCIS) is a Stage 0 pre‐invasive malignant condition that often progresses to invasive oral squamous cell carcinoma. OCIS is a rare condition, with an incidence of just 0.14 per 100,000 people, and it is described as showing histologic features of carcinoma without stromal invasion [5, 6]. OCIS was merged with the severe dysplasia disease classification in 2017 because these conditions could not be distinguished histologically [7]. Despite recognition as a formal histologic diagnosis, some have taken issue with the classification of this condition as “carcinoma” as it has been shown to have a distinct genetic fingerprint [8]. However, the natural course, rate of transformation to invasive cancer, and evidence‐based treatment patterns for OCIS are not well understood [9, 10]. Understanding these clinical characteristics will help better understand OCIS as a distinct disease entity.

OCIS is associated with tobacco and alcohol consumption and, in certain parts of the world, with betel nut chewing. Importantly, these are many of the same risk factors for oral premalignant disorders (OPMD), such as lichen planus, oral lichenoid lesions, leukoplakia, erythroplakia, proliferative verrucous leukoplakia, and oral submucous fibrosis, which transform to OSCC at variable, lower rates when compared to OCIS [11]. However, the similar clinical profile of OCIS, its relative rarity, and its often‐similar appearance may lead OCIS to be managed as an OPMD, resulting in less intensive monitoring or even a lack of treatment altogether [9].

Given a dearth of studies related to OCIS, we performed a large database analysis examining the malignant transformation rates and characteristics of OCIS to OSCC.

## 2 Materials and Methods

### 2.1 Data Source and Study Design

The study data was obtained from the TriNetX Research Network (Cambridge, MA). TriNetX is a global federated health research network providing access to electronic medical records (diagnoses, procedures, medications, laboratory values) from large healthcare organizations (HCOs) [12]. The TriNetX platform only uses aggregated counts and statistical summaries of de‐identified information. No protected health information or personal data is available to platform users. Therefore, this study was deemed exempt by the Penn State University Institutional Review Board (IRB), and the need for informed consent was waived (STUDY18629). This study follows the Strengthening the Reporting of Observational Studies in Epidemiology (STROBE) reporting guidelines for cohort studies.

### 2.2 Participants

The database was queried on January 05, 2025, to identify patients ≥ 18 years of age diagnosed with OCIS between January 01, 2008, and December 31, 2018, allowing for up to 5 years of follow‐up. A control cohort of patients not diagnosed with OCIS or any oral premalignant lesions was generated. ICD‐10‐CM codes for patient inclusion were: D00.00 (carcinoma in situ of oral cavity, unspecified site), D00.01 (carcinoma in situ of labial mucosa and vermilion border), D00.02 (carcinoma in situ of buccal mucosa), D00.03 (carcinoma in situ of gingiva and edentulous alveolar ridge), D00.05 (carcinoma in situ of hard palate), D00.06 (carcinoma in situ of floor of mouth), and D00.07 (carcinoma in situ of the tongue).

### 2.3 Outcome Measures

Patient characteristics, including demographic and lifestyle characteristics, were collected. These included age, race, ethnicity, and lifestyle factors known to contribute to OCIS and oral cavity malignancy: alcohol dependence and nicotine dependence (including both cigarette smoking and chewing tobacco). There is no specific code for betel nut use.

Our primary outcome was the malignant transformation rate of OCIS to invasive oral cavity cancer, defined as ICD‐10‐CM: C00 (malignant neoplasm of lip), C02 (malignant neoplasm of other and unspecified parts of the tongue—not base of tongue), C03 (malignant neoplasm of gum), C04 (malignant neoplasm of floor of mouth), C05.0 (malignant neoplasm of hard palate), and C06 (malignant neoplasm of other and unspecified parts of the mouth). Secondary outcomes included treatment pathways, 5‐year survival probability, and comparison of MP rates to the general population not diagnosed with OCIS.

### 2.4 Statistical Methods

All descriptive and statistical analyses were conducted on the TriNetX platform [13]. Five‐year survival probabilities for OCIS were determined using Kaplan–Meier modeling. The relative risk (RR) of invasive OSCC within 5 years of first diagnosis with OCIS was compared to control patients with no history of OCIS diagnosis. Additional analysis was performed after these two groups were matched according to age to limit confounding. Propensity score matching was conducted via a 1:1 nearest neighbor algorithm using logistic regression. By default, the platform sets a tolerance level of 0.01 and a standard deviation (SD) of 0.1 caliper. *Z* tests were conducted to assess associative differences between our cohort outcomes, and they were reported as RR, 95% confidence intervals (95% CI), and *p* values. Chi^2^ tests, reported as Chi^2^ statistics and *p* values, were used to describe differences in patient lifestyle characteristics. For all statistical tests, significance was set at *p* < 0.05, and all tests were two‐sided. Missing data were handled using pairwise deletion.

## 3 Results

Among 4130 patients diagnosed with OCIS between January 1, 2008, and December 31, 2018, the mean age was 62.5 ± 13.1, with patients being 61.0% male (*n* = 2521), 74.0% white (*n* = 3058), and 73.3% not Hispanic/Latino (*n* = 175). The mean annual incidence rate of OCIS diagnosis was 0.28 per 100,000 person‐years. Patients with OCIS had overall rates of 15.3% nicotine dependence (*n* = 15.3) and 7.5% alcohol dependence (*n* = 308) (Table 1).

**TABLE 1:** Population demographics and lifestyle characteristics of patients diagnosed with OCIS between 2008 and 2018.

Cohort total	4130
Annual diagnoses, *n* (%)	357.5 ± 161.6
Diagnosis rate per 100,000 person‐years	0.28
Age	62.5 ± 13.1
Sex, *n* (%)	
Male	2521 (61.0)
Female	1524 (36.9)
Race, *n* (%)	
White	3058 (74.0)
Black or African American	288 (7.0)
Asian	159 (3.9)
Native Hawaiian or other Pacific Islander	37 (0.9)
Native American or Alaskan	11 (0.3)
Other	80 (1.9)
Unknown	497 (12.0)
Ethnicity, *n* (%)	
Hispanic or Latino	175 (4.2)
Not Hispanic or Latino	3028 (73.3)
Unknown	927 (22.4)
Lifestyle characteristics, *n* (%)	
Oral submucous fibrosis	22 (0.5)
Nicotine dependence	632 (15.3)
Alcohol dependence	308 (7.5)

**FIGURE 1:**
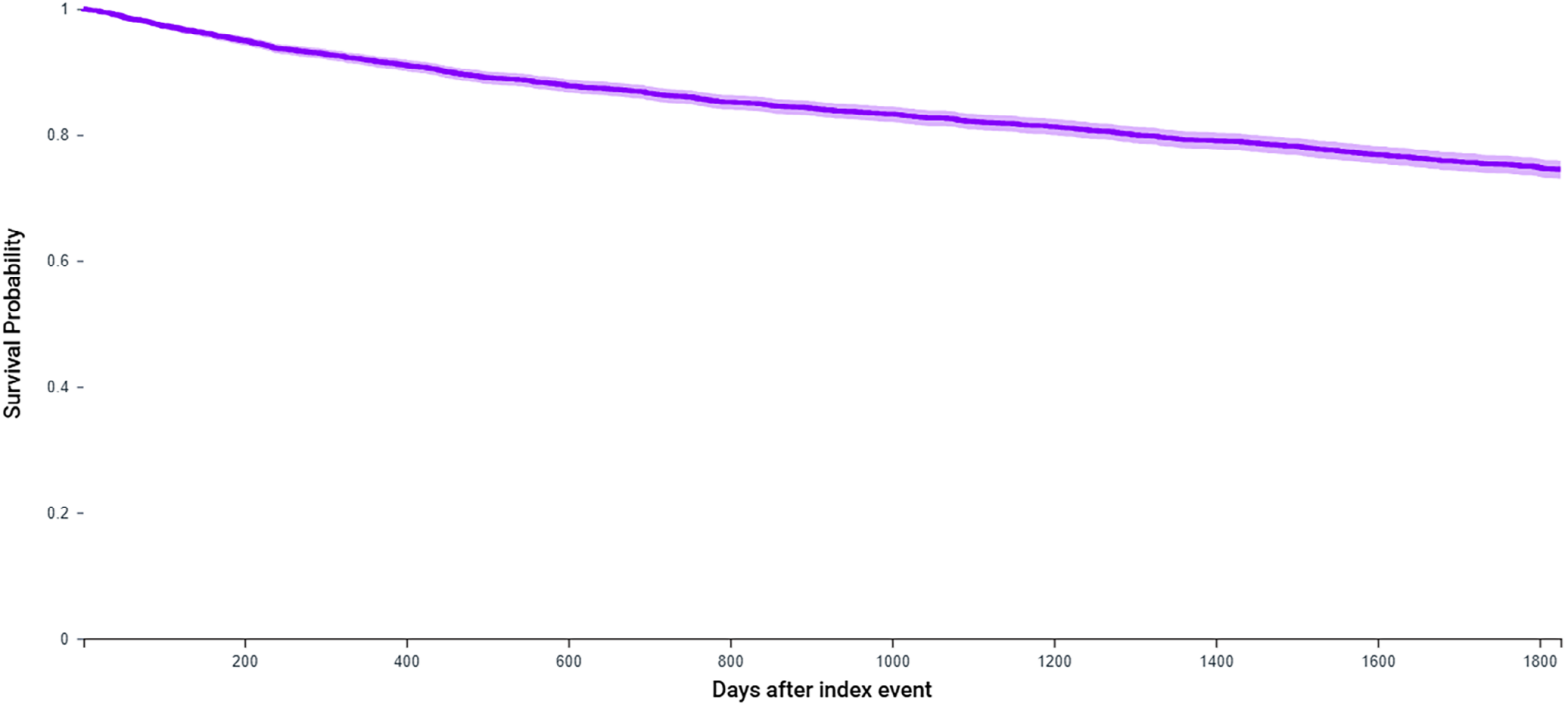
Kaplan–Meier survival curve demonstrating survival probability over a 5‐year period following OCIS diagnosis. [Color figure can be viewed in the online issue, which is available at www.laryngoscope.com]

The overall 5‐year malignant transformation rate from OCIS to invasive OSCC was 56.8% (RR 234.9 [126.4–436.5 95% CI] *p* < 0.01 vs. control), with the mean time to transformation being 15.0 ± 2.2 months. The survival probability for OCIS was 74.7% (Table 2, Figure 1).

**TABLE 2:** Malignant transformation rates, treatment patterns, and 5‐year survival probability of patients diagnosed with OCIS between 2008 and 2018.

MT rate to OSCC, *n* (%)	2349 (56.8)
RR (95% CI) vs. control	234.9 (126.4–436.5)
*p* value versus control	< 0.01
Time to MT	15.0 ± 2.2
Treatment, *n* (%)	2135 (51.7)
Excisional surgery	1008 (24.4)
Chemo‐Radiation Therapy	1521 (36.8)
5‐year survival probability (%)	74.7

Among patients with OCIS, 51.7% (*n* = 2135) had an excisional surgical, chemotherapeutic, or radiotherapeutic intervention within 5 years of diagnosis (RR 7.0, 6.2–7.8 95% CI, *p* < 0.01 compared to matched control cohort). Excisional surgery was carried out for 24.4% (*n* = 1008) of patients, while 36.8% (*n* = 1521) underwent chemo‐radiation therapy within 5 years of OCIS diagnosis. Excisional therapy was first‐line in 39.4% of treated patients, most commonly followed by chemotherapy or radiation (at similar levels) as second‐line treatment. Chemotherapy was first line for 33.2% of patients, and radiation for 21.0%. About 48.0% of patients in our study cohort only underwent watchful waiting as first‐line treatment.

## 4 Discussion

This study aimed to characterize the 5‐year malignant transformation rate of OCIS to squamous cell carcinoma using a retrospective large database analysis. We found a 5‐year malignant transformation rate of 56.8% among 4130 patients diagnosed with OCIS over 10 years. This study represents the largest single analysis investigating the malignant transformation rate of OCIS.

Early recognition and diagnosis of OCIS might improve patient survival and reduce treatment‐related morbidity. The gold standard for diagnosing oral lesions with malignant potential remains comprehensive history‐taking, physical examination of the oral cavity, and biopsy [14]. Population‐based screening programs for OCIS are expensive due to the low prevalence of lesions in the general population of developed countries [14, 15]. While screenings conducted by professionals are more accurate, they are also more costly than those performed by healthcare auxiliaries. However, consensus regarding screening guidelines has remained persistently contentious. The most recent recommendation statement from the United States Preventative Services Task Force (USPSTF) from 2013 found that “current evidence is insufficient to assess the balance of benefits and harms of screening for oral cavity cancer in asymptomatic adults.” [15] Based on data up to 2011, this recommendation excludes otolaryngologists and dentists and encourages these professionals to conduct comprehensive oral cavity exams during encounters [15]. No further statements have been made since 2013. Conversely, the American Head and Neck Society (AHNS) has taken a distinct stance, advocating for comprehensive oral head and neck exams for individuals with concerning symptomology or those with risk factors, as OPMD and early‐stage oral cancers may present asymptomatically [16].

The consensus regarding treatment is also unsettled. Most comprehensive studies of OCIS date back to the latter half of the 20th century, and they find that lesions with *any* degree of dysplasia have a 12.1% risk for malignant transformation in a mean of 4.3 years [17]. It is this high potential for malignant transformation that led the AHNS to recommend excision of lesions with high‐grade dysplasia or those diagnosed as OCIS [16]. This is similar to the OPMD literature, which suggests excision or ablation and regular lifetime monitoring of OPMD demonstrating high‐grade dysplasia [3, 4]. With an elevated 5‐year malignant transformation rate of 56.8% in a mean of just 15.0 months, our study highlights an additional risk with the current management paradigm: undertreatment of OCIS after diagnosis. Additionally, our survival probability rate of just 74.7% approaches the 69.6% age‐adjusted overall survival rate of OSCC, further highlighting potential mismanagement of OCIS by diagnosing clinicians [18]. Based on the findings of our study and in agreement with AHNS guidelines, we recommend prompt excision of any OCIS lesions with clear margins and close follow‐up. Prompt recognition, diagnosis, and treatment of OCIS are thus necessary to mitigate transformation to OSCC and the quality of life and survival impacts of more advanced malignancy.

As mentioned, documentation of the rate of OCIS transformation is sparse in the literature, with the most well‐cited publications coming from over 40 years ago [19, 20, 21]. We present a study using more recent data. We also use specific coding for OCIS and a follow‐up period of up to 5 years following diagnosis, which is not always feasible in prospective or retrospective institutional studies.

Despite the strengths and findings of our investigation, important limitations exist within the context of our study question and design. While the TriNetX network is expansive, there is limited data related to patient follow‐up within HCOs included in the database versus treatment at outside institutions. This could introduce bias in our results, as patients may have been diagnosed with OCIS at an outside institution before receiving specialty care at a larger HCO within the TriNetX network. Further, there is limited lifestyle data recorded in this database outside of ICD‐10 codes, which was especially pertinent in our presentation of low proportions of included patients with nicotine and alcohol usage. Additionally, as with all large electronic medical record‐sourced database studies, our analysis is limited by the accuracy of data entry. This may contribute to undercounts of OCIS or lifestyle factors such as tobacco or alcohol use. Furthermore, despite efforts to control for demographic characteristics in our analyses, there could be additional influences of confounders not measured or available within our database, such as genetic predispositions, environmental exposures, or effects of medications.

## 5 Conclusions

We describe the largest study to date characterizing the rate of malignant transformation of OCIS to invasive OSCC. Our results indicate a high rate of malignant transformation of OCIS to invasive OSCC over 5 years and a poor 5‐year survival probability of just 74.7%. OCIS needs to be differentiated from oral premalignant diseases when seen in primary care or in general dental practice and referred for pathologic and specialist prompt work‐up to mitigate or prevent transformation of OCIS to invasive oral cavity cancer. Future studies should seek to better understand characteristics of disease transformation and work to improve awareness among treating physicians.

## Disclosure

This article was presented at the Triological Society 2025 Combined Sections Meeting, January 23–25, 2025, in Orlando, FL.

## Conflicts of Interest

The authors declare no conflicts of interest.
